# COVID-19 severity detection using chest X-ray segmentation and deep learning

**DOI:** 10.1038/s41598-024-70801-z

**Published:** 2024-08-27

**Authors:** Tinku Singh, Suryanshi Mishra, Riya Kalra, Satakshi, Manish Kumar, Taehong Kim

**Affiliations:** 1 School of Information and Communication Engineering, Chungbuk National University, Cheongju, South Korea; 2 Department of Mathematics & Statistics, SHUATS, Prayagraj, Uttar Pradesh India; 3 Indian Institute of Information Technology Allahabad, Prayagraj, Uttar Pradesh India

**Keywords:** COVID-19, Deep learning, Brixia score, Capsule network, Chest X-ray, Infectious diseases, Computer science, Information technology

## Abstract

COVID-19 has resulted in a significant global impact on health, the economy, education, and daily life. The disease can range from mild to severe, with individuals over 65 or those with underlying medical conditions being more susceptible to severe illness. Early testing and isolation are vital due to the virus’s variable incubation period. Chest radiographs (CXR) have gained importance as a diagnostic tool due to their efficiency and reduced radiation exposure compared to CT scans. However, the sensitivity of CXR in detecting COVID-19 may be lower. This paper introduces a deep learning framework for accurate COVID-19 classification and severity prediction using CXR images. U-Net is used for lung segmentation, achieving a precision of 0.9924. Classification is performed using a Convulation-capsule network, with high true positive rates of 86% for COVID-19, 93% for pneumonia, and 85% for normal cases. Severity assessment employs ResNet50, VGG-16, and DenseNet201, with DenseNet201 showing superior accuracy. Empirical results, validated with 95% confidence intervals, confirm the framework’s reliability and robustness. This integration of advanced deep learning techniques with radiological imaging enhances early detection and severity assessment, improving patient management and resource allocation in clinical settings.

## Introduction

Coronavirus disease (COVID-19) is an infectious disease caused by the SARS-CoV-2 virus, and it enters into the human body through the mouth, nose, or eyes^1^ (directly from airborne droplets or virus transfer through hands to the face). Once inside the nasal passages, it travels to the mucous membrane at the back of the throat. In addition to attaching to cells, it multiplies and enters lung tissue. Once in the body, the virus is capable of spreading to other parts of the body. Most people infected with the virus will have mild to moderate respiratory illness and recover without special treatment. Some, however, will become critically ill and require medical attention. The disease severely impacts people because it affects the lungs and causes severe breathing problems and lung infections^2^. People over the age of 65, as well as those with underlying medical conditions such as cardiovascular disease, diabetes, chronic respiratory disease, or cancer, are at a higher risk of developing serious illnesses. However, any age group can become seriously ill or die from COVID-19^3^. Early detection is vital for patient care and protecting community health by ensuring prompt patient isolation. One of the major challenges in managing COVID-19 is differentiating its symptoms from other respiratory illnesses like pneumonia, as they may present similarly. Additionally, a negative test result does not rule out infection, especially for individuals with known exposures. While real-time polymerase chain reaction (RT-PCR) testing for antigens is accurate, it can be time-consuming and limited by testing resources and laboratory equipment. Considering the drawbacks of chest computed tomography (CT) scans, such as higher costs and adverse radiological effects, chest radiographs (CXR) are a more suitable alternative for efficient testing, conserving time and resources. While CXR is less costly and minimizes radiation exposure compared to CT scans, its sensitivity in detecting COVID-19 may be lower. Current methods, such as RT-PCR, face limitations in terms of availability, cost, and time required for results. CXR imaging presents a viable alternative but requires enhanced techniques to improve diagnostic accuracy and provide insights into the severity of lung infections.

Machine learning (ML) can be employed in medical diagnosis, particularly in domains like radiology, cardiology, and oncology. It helps in analyzing complex medical data, which helps in boosting the accuracy and speed of diagnosis. Additionally, ML also plays a significant role in diagnosing and screening critical conditions of the patients^4^. In the context of diseases like COVID-19, ML and Deep Learning (DL) prove invaluable in identifying potential causes and symptoms. Leveraging medical imaging data such as chest radiography or CT scans, these technologies aid in early detection and diagnosis^4,5^. Convolutional Neural Networks (CNN), a popular DL architecture for image analysis, have gained recognition in the context of COVID-19 diagnosis due to their ability to analyze medical imaging data. Researchers have developed CNN-based models trained on large datasets of CXR images, aiming to distinguish COVID-19 cases from other respiratory conditions or normal cases^6,7^. Alongside CNN, capsule networks have emerged as a promising alternative for COVID-19 diagnosis^8^. ML and DL techniques have shown promise in medical imaging applications, including the detection of COVID-19, but there is a need to develop robust frameworks that can accurately classify the infection and assess its severity.

This paper presents a comprehensive framework for detecting COVID-19 infections in CXR images and facilitating the categorization of these CXR images into different levels of lung infection severity, including normal, mild, moderate, and severe.The categorization relies on the Brixia score, a clinical grading system designed to assess the severity of lung infections in hospitalized COVID-19 patients^9^. Higher Brixia scores correlate with increased inpatient mortality rates. The framework starts with the U-Net model^10^ for precise segmentation of CXR images. Next, a capsule network processes the segmented lung images, classifying them into normal, pneumonia, or COVID-19 categories. Several advancements in the design and implementation of the classifier and U-Net model are presented. The proposed model integrates the strength of capsule networks with the U-Net architecture to enhance feature representation and segmentation accuracy. For COVID-19 cases, the Brixia score is used to assess infection severity. To improve the accuracy of severity classification, DL models such as ResNet50^11^, VGG-16^12^, and DenseNet201^13^ are employed. This demonstrates the framework’s robustness and effectiveness in identifying subtle variations in severity levels. This paper makes the following contributions:

- Providing multi-level (classification and severity detection) of CXR images: the first stage utilizes U-Net for precise image segmentation, followed by a capsule network to classify CXR images into normal, pneumonia, or COVID-19 categories.
- In the second stage, the severity of the infection is evaluated, and COVID-19 detected images are further classified into normal, mild, moderate, and severe categories using the Brixia score methodology.
- It facilitates early detection of COVID-19 and provides detailed information about the severity of infections, which is crucial for appropriate patient care and management.
- The effectiveness of state-of-the-art DL models in severity classification is compared, enhancing the robustness and reliability of the framework.

The rest of the paper is arranged in the following manner: Section “Literature review” presents an overview of the work done to date in this area. Section “Methodology” explains the proposed methodology and implementation details. Details about distinct performance metrics are outlined in section “Performance evaluating measures”. Section “Dataset” delves into specific details about the dataset. Experimental configurations and results are discussed in section “Experimental results and discussion”. Finally, the paper’s conclusion and its future direction are covered in section “Conclusion and future work”.

## Literature review

A variety of techniques have been developed by researchers to identify the COVID-19 virus during the crisis, utilizing datasets containing symptom information, CT scans, and CXR for virus detection. Technological advances in ML and DL have significantly enhanced various medical imaging applications. Studies^14–17^ have demonstrated the potential of patch-based DL approaches for improving classification and segmentation accuracy in medical images, including breast cancer, CT vertebrae, and liver segmentation tasks. These studies highlight the effectiveness of deep belief networks, overlapping patches, and lightweight CNN, achieving high accuracy and precision across different datasets despite challenges like computational complexity and the need for extensive labeled data.

To leverage existing infrastructure^18^, researchers have proposed a COVID-19 patient screening approach based on the results of CT and X-ray examinations. Early studies have demonstrated relatively accurate disease diagnosis using ML and DL methods. For instance, Pritam Saha et al.^19^ introduced GraphCovidNet, a Graph Isomorphic Network (GIN) model, to detect COVID-19 from CT scans and CXRs of affected patients. Rezaeijo et al.^20^ focused on automatic prediction of COVID-19 using deep transfer learning models and ML algorithms applied to chest CT images, with the DenseNet201 model and KNN algorithm showing superior performance when combined with pre-trained models. Khan et al.^21^ introduced a two-phase deep CNN framework, incorporating SB-STM-BRNet and COVID-CB-RESeg, which addresses challenges such as limited labeled data and high structural similarity in lung CT images. This method demonstrates high accuracy in detecting and segmenting COVID-19 infections. However, the framework’s reliance on extensive training data and computational resources poses limitations. Additionally, Heidarian et al.^22^ proposed a fully automated framework based on capsule networks to identify COVID-19 cases from chest CT scans, demonstrating the superiority of capsule networks in terms of trainable parameters and accuracy compared to CNN-based alternatives. Another alternative, the COVID-CAPS model^8^, also based on capsule networks, was suggested by Afsar et al., capable of handling small datasets, which is crucial given the sudden and rapid emergence of COVID-19. Ter-Sarkisov et al.^23^ developed a COVID-19 prediction method based on CT scans using regional features, achieving an impressive overall accuracy of 91.66% on the test data. These models still face difficulties in generalizing across different datasets and imaging conditions.

CT scans have shown superior sensitivity and specificity in diagnosing COVID-19 compared to CXR^24^. However, it is crucial to note that CT scans expose patients to 70 times more radiation than CXR, slightly increasing the risk of cancer due to medical radiation exposure^25^. As a result, researchers have explored classical image processing methods and machine/deep learning approaches to automatically classify diseases using digitized CXR^26,27^. Rahul Kumar et al.^28^ proposed a framework for accurate COVID-19 prediction using deep feature learning with SMOTE and ML classifiers, training on CXR images using the ResNet152 architecture. Meanwhile, Ghaderzadeh et al.^29^ conducted a review study providing an overview of current models for detecting and diagnosing COVID-19 using DL with radiology modalities. They emphasized the importance of avoiding overfitting and maximizing the generalizability and usefulness of COVID-19 DL diagnostic models by training them on large, diverse datasets covering the entire available data space. Studies^30–32^ introduced several DL approaches for detecting COVID-19 from CXR images. A channel-boosted CNN showed improved detection accuracy using auxiliary channels generated through transfer learning^30^. The COVID-RENet-1 and COVID-RENet-2 architectures utilized region and edge-based operations to capture pneumonia-specific patterns, achieving high F-scores and accuracy^31^. The Deep Boosted Hybrid Learning framework combined these models’ strengths through feature space boosting, resulting in excellent performance and a web-based interface for rapid COVID-19 detection^32^. However, these models require substantial computational resources and may struggle to generalize across different datasets or imaging conditions. Additionally, their reliance on high-quality labeled data can limit performance due to variations in image acquisition and labeling standards.

**Table 1:** Comparison of different approaches studied in the literature review.

S.No.	Method name	Empirical results	Strengths	Weaknesses
1	Patch-based DL approach^14–17^	Improved classification and segmentation accuracy	High accuracy and precision across datasets	Computational complexity, need for extensive labeled data
2	COVID-19 detection models^21,33^	High accuracy in detecting and segmenting COVID-19 infections	Advanced techniques like STM blocks and FME	Limited labeled data, high computational complexity
3	Graph-based and transfer learning models^19,20^	Effective COVID-19 detection and prediction	Utilizes GIN and transfer learning models	Dependence on large datasets for training
4	Capsule networks^8,22^	Superior performance with small datasets	Better handling of small datasets	Complex architecture
5	Regional feature-based prediction^23^	Overall accuracy of 91.66% on test data	Effective use of regional features	Limited generalizability
6	Deep feature learning with SMOTE^28^	Accurate COVID-19 prediction using CXR images	Improved accuracy with ResNet152 architecture	Potential overfitting
7	Ensemble and hybrid learning models^30–32,34^	High performance with web-based interface for rapid detection	Combines strengths of multiple models	Substantial computational resources required
8	Severity assessment models^35–38^	Efficient and reliable assessment of COVID-19 severity	Accurate severity computation	Complex preprocessing and segmentation steps

Identifying COVID-19 in individuals is essential, but assessing the severity of the disease and its impact on the lungs is equally critical for understanding disease progression and hospital resource management^39,40^. Studies have shown a strong correlation between COVID-19 severity and factors like ICU admissions, hospital stays, and follow-up planning^41,42^. To efficiently and reliably assess COVID-19 severity in patients, Li et al.^35^ utilized CXR, employing the Brixia score for severity computation and U-Net++ for lung-related CXR image segmentation. Zandehshahvar et al.^36^ utilized a CNN model to classify COVID-19 CXR into severity levels (normal, mild, moderate, severe). Shelke et al.^37^ proposed a framework that classifies CXR into four categories: normal, tuberculosis, pneumonia, and COVID-19, while also assessing COVID-19 severity. Another study by Cohen et al.^38^ evaluated COVID-19 pneumonia severity on CXR using scores from blinded experts. Udristoiu et al.^34^ employed an ensemble of DL and pre-trained models, achieving impressive results for all diagnosis classes. In an acute COVID-19 outbreak, CXR analysis^43^ revealed that the severity of opacities was related to advanced age, comorbidities, and acuity of care, highlighting the feasibility of artificial intelligence tools based on DL for assessing COVID-19 CXR during outbreaks.

As indicated in the literature review (see Table 1), DL models can effectively detect COVID-19 when applied alongside CT scans and CXR. These methods work by identifying image features, starting with simple attributes like edges and progressing through layers to more complex ones. Additionally, the studies^34–38,43^ demonstrate that CXR images can be used to assess the severity of lung conditions. However, there has been limited attention on utilizing DL methods with CXR images to assess the extent of organ impact caused by COVID-19, particularly in the case of organs like the lungs. DL models primarily focus on detecting features in images without considering their spatial arrangement. Consequently, they may overlook spatial information and incorrectly select certain features, leading to inaccurate predictions and increased costs. To tackle this challenge, we propose utilizing the capsule network, which not only captures features but also considers their spatial relationships. Developing predictive models for images presents its own set of challenges, including accurate data labeling and strong inter-rater agreement. Furthermore, creating a representation that generalizes to new images becomes particularly complex when the available labeled images are scarce. This becomes especially evident when developing a predictive tool for COVID-19 CXR images, as the absence of a publicly accessible dataset complicates the evaluation process.

## Methodology

This study presents a framework aimed at the classification and severity assessment of COVID-19-infected individuals. The foundation of this process rests upon the analysis of CXR images, capturing the spatial extent of viral proliferation. To accomplish this, the U-Net architecture is harnessed, functioning as a generator of segmentation masks designed to delineate lung regions within the X-ray data.

## Dataset

The COVID-19 Radiography Dataset employed in this study^44^ is a publicly available dataset developed by a team from Qatar University, Dhaka University, Bangladesh with cooperators from Malaysia and Pakistan and cooperators of medical doctors. It comprises 3616 entries for COVID-19, 1345 entries for viral pneumonia, 10192 entries for normal cases, and 6012 entries for infections other than COVID-19 and the corresponding lung masks. These images are then added to the data frame with corresponding labels: 0 for COVID-19, 1 for viral pneumonia, and 2 for normal cases. The dimensions of all images are checked and confirmed to be $(299 \times 299 \times 3)$, eliminating resizing. Next, the images are transformed into arrays of feature-scaled pixels by dividing each pixel value by 255. Additionally, the images’ labels are changed into vectors where 0 is represented as [1 0 0], 1 as [0 1 0], and 2 as [0 0 1].

## Methods

**Figure 1:**
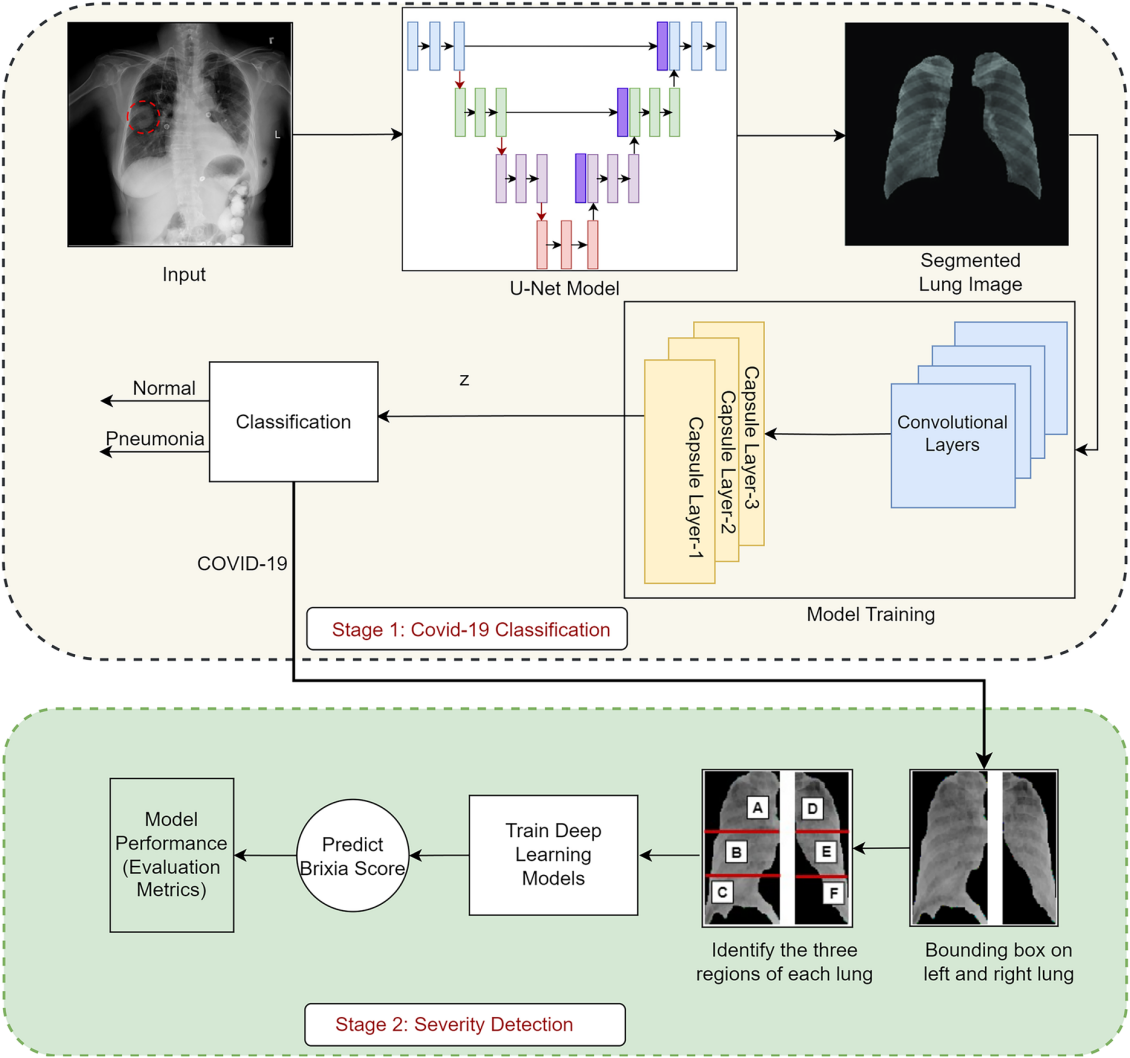
Proposed framework.

The CXR scans are provided as input into the U-Net framework, leading to the creation of lung-specific segmented masks as illustrated in Figure 1. This accomplishment is derived from the incorporation of a specialized biomedical U-Net architecture. A dedicated segmentation phase is integrated into the proposed model, elevating diagnostic efficiency through rapid and precise analyses. The resultant segmented CXR scans are utilized for classification and severity assessment, respectively, offering a comprehensive approach to the diagnostic challenge. CXR images are classified through the CNN, targeting specific dimensions for insights. To extract lung segmentation information from X-ray images exhibiting infection, an artificial neural network leverages a CNN architecture. Each X-ray image of the segmented lung segment undergoes a process through a series of convolution layers, pooling, fully connected layers, culminating in the application of the softmax function for precise data classification. The CNN model disregards feature location, concentrating solely on detected features. Therefore capsule networks, which is capable of extracting spatial information and crucial attributes, have been employed to mitigate information loss during pooling operations. Moreover, segmented lung images are categorized into three groups based on different diseases for accurate classification. If individuals are either unaffected or diagnosed with pneumonia after classification of the segmented CXR scans, no further action is taken.

However, those presenting COVID-19 symptoms undergo additional testing. The collected lung images undergo segmentation using the U-Net model, combined with a classification technique, to establish the severity of infection in each affected individual. Contours are extracted from the mask image utilizing a segmentation mask and subsequently sorted by area. As segmented lung images prominently display lung region contours, based on these segmented images both the left and right lung image areas are divided and cropped accordingly. The Brixia scoring system is applied to meticulously analyze variations in CXR, segregating the segmented mask (both left and right) into three distinct vertical groups, each with equal area proportions. Leveraging DL models such as ResNet50, VGG-16, and DenseNet201, rigorous fine-tuning endeavors aim to estimate the Brixia score accurately. Moreover, the model’s performance has been evaluated using the performance evaluating measures discussed in section “Methods”. The following subsections provide a comprehensive explanation of the methods employed within the proposed framework.

### Convolution layers and capsule networks (Conv-Caps)

Convolution layers utilize various convolution kernels to extract features from segmented CXR images. The capsule networks evaluate the likelihood of specific objects being present, incorporating multiple neurons representing diverse instantiation characteristics like rotation and size, all linked to the underlying objects. To mitigate issues associated with pooling layers in CNN, these layers are substituted with the “routing by agreement” technique. Instead of straightforwardly subsampling the feature maps, the contribution of each capsule is assessed by its ability to predict subsequent capsules’ outputs, as opposed to recklessly sub-sampling the feature maps. Consider that the $r\textrm{th}$ convolutional kernels employ the *M* layers. In the $(M+1)$ layer, the $j\textrm{th}, j = (1, 2,..., r),$ convolutional feature map can be represented as:

1 $$\begin{aligned} \mathscr {T}^{M+1}_{j} = f \left( \sum _{p} \mathscr {U}_{j}* \mathscr {T}^{M}_{p}\right) \end{aligned}$$

In Eq. (1) $\mathscr {T}_{p}$ is the feature map of the $m\textrm{th}$ kernels. The $\mathscr {U}_{j}$ is the $j\textrm{th}$ kernel while as $T_{p}$ may be a channel of the actual image of the CXR, a convolutional map, or a pooling map, *f* (.) specifies a nonlinear activation function, while $*$ denotes the convolution operation. In CNN, the rectified linear unit (ReLU) is frequently used with the non-linear function $J(y) = max(0,y)$. The input images of segmented lungs are frequently compressed in size using a pooling technique. The pooling map in layer $(M+1)$ is generated by conducting a pooling operation on the relevant features in layer *M* previously and is provided as follows:

$$\begin{aligned} \mathscr {T}^{M+1}_{j} = pool \left( \mathscr {T}^{M}_{j}\right) \end{aligned}$$

where pool(.) denotes the pooling strategy and index *j* traverses each map in layer *M*.

**Figure 2:**
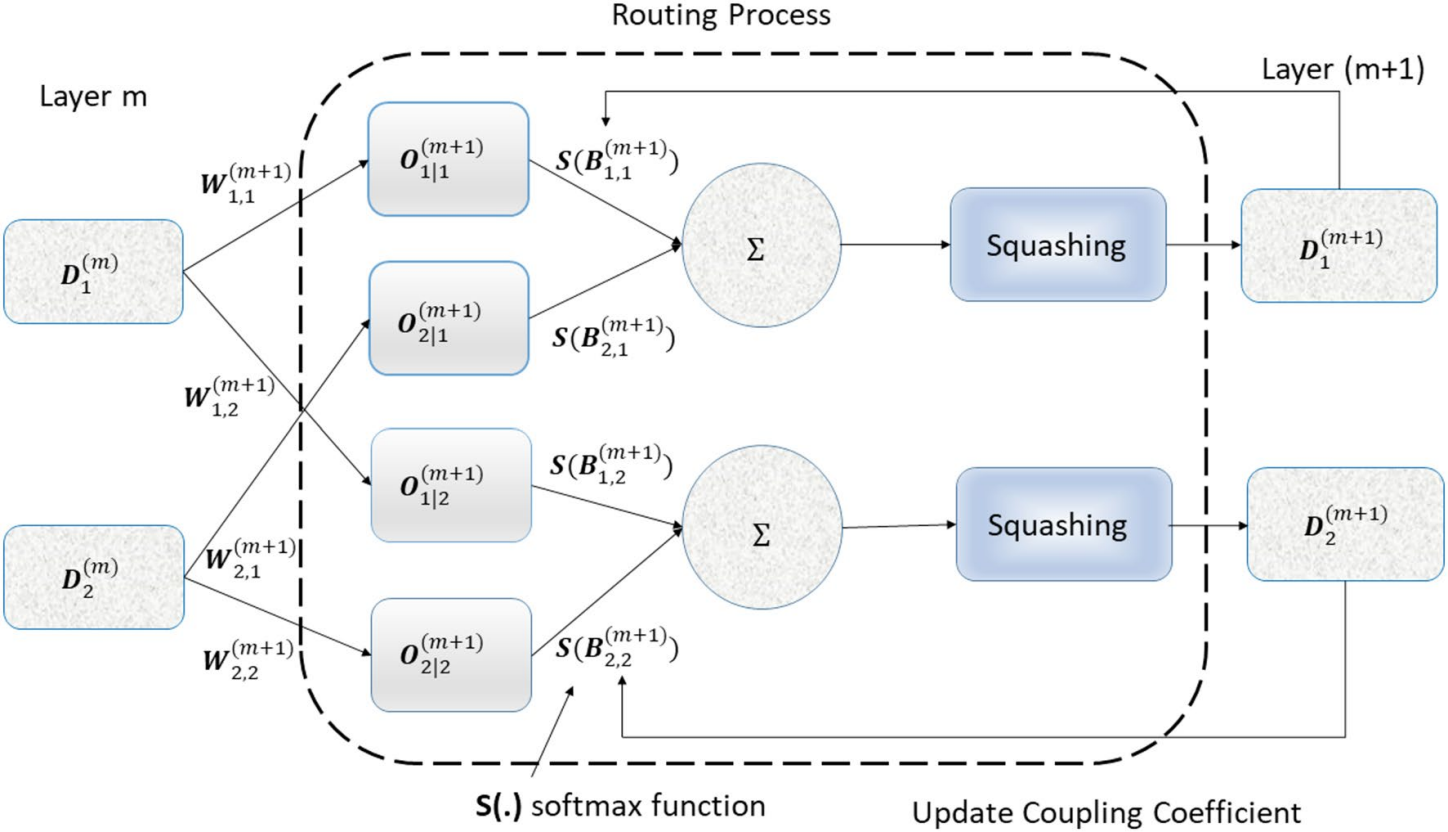
Routing coefficient process.

In general, the most common kind of connection between layers is the scalar-scalar form in CNN. A capsule network^45^ consists of a group of neurons representing an entire or portion of an entity. The Conv-Caps model employs capsule layers instead of traditional convolutional layers, which capture spatial relationships and provide a more nuanced feature representation vital for distinguishing between classes. Capsule networks use a dynamic routing mechanism that iteratively adjusts the weights of connections between capsules, enhancing the robustness of the network by ensuring that important features are dynamically weighted more heavily. We incorporate both max pooling and average pooling operations in our network, improving the diversity of the feature space and enhancing the classifier’s ability to generalize across different datasets. Neurons are replaced by groups of neurons, and the capsule layers are interconnected in vector-vector form. The output of each capsule in a layer is modeled as an attempt to predict the output of a capsule in the layer above it. If its prediction matches the actual output, the connection between them is reinforced. This iterative process ensures that capsules with more accurate predictions have a greater impact on the network’s final decision, ultimately improving the network’s ability to capture complex spatial relationships in the data. The following nonlinear squash function $\mathscr {F}{{\textbf {(z)}}}$ is described for every capsule (expressed as a vector):

2 $$\begin{aligned} \mathscr {F}{{\textbf {(z)}}} = \frac{\Vert {\textbf {z}} \Vert ^{2}_{2}}{1+ \Vert {\textbf {z}} \Vert ^{2}_{2}} \cdot \frac{{\textbf {z}}}{\Vert {\textbf {z}} \Vert _{2}} \end{aligned}$$

where $\Vert \cdot \Vert _{2}$ stands for the $\ell _{2}$-norm, and ${\textbf {z}}$ is the squash function’s input vector in Eq. (2). With the help of this function, short vectors’ lengths are reduced to almost zero, while long vectors’ lengths are nearly one. Consequently, the entity’s probability can be represented by its output length. The $i^{th}$ capsule’s output, ${\textbf {u}}_{i}$, is determined by:

3 $$\begin{aligned} {\textbf {u}}_{i} = \mathscr {F}({\textbf {w}}_{i}) \end{aligned}$$

where ${\textbf {w}}_{i}$ stands for the input of the $i\textrm{th}$ capsule. Each capsule’s parameters are associated with various properties, including its position, scale, and orientation. A weighted sum is employed for the overall input ${\textbf {w}}_{i}$ of the $i\textrm{th}$ of all capsules except the first capsule layer:

$$\begin{aligned} {{\textbf {w}}}_{i} = \sum _{n} A_{ni} {{\textbf {O}}}_{i\vert n} \end{aligned}$$

where ${{\textbf {O}}}_{i\vert n}$ is the estimated output of capsule *i* in the current layer based on capsule *n* from the preceding layer, and $A_{n,i}$ is the process of routing coefficient represented in Fig. 2. Assume that ${\textbf {B}}_{ni}$ refers to the log prior probability that capsule *n* (from the preceding layer) is coupled with capsule *i* (from the present layer). Hence, the coefficients $A_{ni}$ may be written as:

4 $$\begin{aligned} A_{ni} = \frac{exp ({\textbf {B}}_{ni})}{\sum _{q} {exp ({\textbf {B}}_{nq})}} \end{aligned}$$

In Eq. (4), index *q* represents the all capsule in the current layer. The routing algorithm initializes ${\textbf {B}}_{ni}$ with zeros and updates it. The following procedure updates ${\textbf {B}}_{ni}$ in the routing algorithm:

5 $$\begin{aligned} {\textbf {B}}^{(m+1)}_{ni} = {\textbf {B}}^{m}_{ni} + \langle {\textbf {u}}_{i}, {\textbf {O}}_{i\vert n} \rangle \end{aligned}$$

where *m* represents the iteration index of the layer. The inner product of $\langle u_{i}, {\textbf {O}}_{i\vert n} \rangle$ between the estimated output and its real (actual) output (for capsule *i* in the current layer). Each capsule from the preceding layer will forecast the capsule $i's$ value in the current layer, which is intuitive. Accordingly, if the estimation made by capsule *n* from the preceding layer is analogous to the real output ${\textbf {u}}_{i}$, and capsule *n* has a high probability of contributing, the coupling coefficient $A_{ni}$ increases. Based on Eqs. (3) and (5), output capsules ${\textbf {P}}_{n}$ from the preceding layer, the estimations ${\textbf {O}}_{i \vert n}$ can be evaluated as:

6 $$\begin{aligned} {\textbf {O}}_{i \vert n} = \mathscr {W}_{in} {\textbf {P}}_{n} \end{aligned}$$

where $\mathscr {W}_{i,n}$ stands for the weight transformation matrix linked capsules across two adjacent layers. Assuming that there are $\textbf{Z}$ classes, the last capsule layer consists of $\textbf{Z}$ capsules, whose length represents the existence probability of each object in the class. An X-ray image of the chest that has multiple classes can be segmented using a margin loss function. The margin loss function $\mathscr {L}_{k}$ in Conv-Caps for class $k = (1,2,..., \textbf{Z})$ is defined as:

$$\begin{aligned} \begin{aligned} \mathscr {L}_{k}&= \textbf{U}_{k} \max \left( 0, \textbf{g}^{+} - \Vert \textbf{h}_{k} \Vert _{2} \right) ^{2} \\&\quad + \eta (1 - \textbf{U}_{k}) \max \left( 0, \Vert \textbf{h}_{k} \Vert _{2} - \textbf{g}^{-} \right) ^{2} \end{aligned} \end{aligned}$$

where $\textbf{U}_{k}$ is an indicator function. It can be defined as:

$$\begin{aligned} \textbf{U}_{k}= \left\{ \begin{array}{ll} 1, & If \hspace{0.5mm}the \hspace{0.5mm} sample\hspace{0.5mm} contains\hspace{0.5mm} instances \hspace{0.5mm}of\hspace{0.5mm} class\hspace{0.5mm} k \\ 0, & Otherwise \\ \end{array} \right. \end{aligned}$$

where $\Vert \textbf{h}_{k} \Vert _{2}$ stands for the length of the vector, and $\textbf{h}_{k}$ is the last layer of the capsule. If and only if class k object exists, then sample class label ${\textbf {U}}_{k} = 1$. If objects of class *k* are present, the span (length) of capsule ${\textbf {h}}_{k}$ extends beyond ${\textbf {g}}^{+}$. When there is no object of class *k*, the span of capsule ${\textbf {h}}_{k}$ must be less than ${\textbf {g}}^{-}$. The upper and lower boundary is defined by the boundary parameters ${\textbf {g}}^{+}$ and ${\textbf {g}}^{-}$, respectively. The regularization parameter $\eta$ serves the purpose of shrinking the impact of the activity vector in cases where the represented class is absent from the sample. The $\mathscr {L}_{CLA} = \sum _{k} \mathscr {L}_{k}$ is used to determine the loss function’s entire classification, which reduces the total loss from all last layer capsules. The class prediction thresholds, ${\textbf {g}}^{+}$, and ${\textbf {g}}^{-}$, with values of 0.9 and 0.1 appropriately, are utilized to regulate the class response of the output that has been computed. In particular, $\mathscr {L}_{k} = 0$ if the class capsule layer’s prediction vector ${\textbf {h}}_{k}$ matches ${\textbf {U}}_{k}$.

**Figure 3:**
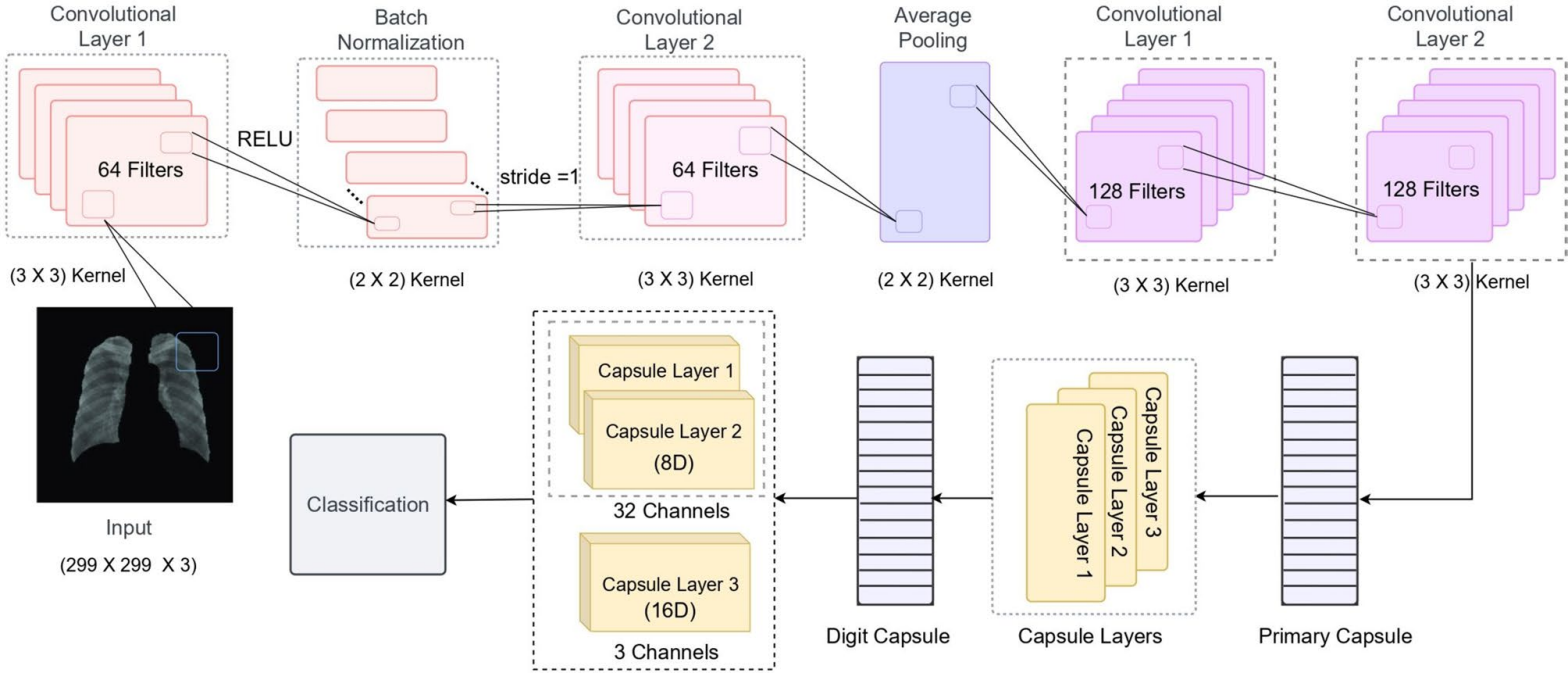
Architecture of capsule network.

An overview of the capsule network architecture is shown in Fig. 3. The initial layer of the primary capsule layer includes a convolutional layer with 64 filters, using a $(3 \times 3)$ kernel size, stride 1, and activating RELU. This layer is followed by another convolutional layer with 64 filters of $(3 \times 3)$ kernel size, stride 1, and activation RELU. After batch normalization, an average pooling layer with a $(2 \times 2)$ kernel size is adopted. In this study, the architecture also incorporates two convolutional layers, each using 128 filters of $(3 \times 3)$ kernel size, stride 1, and activation RELU. Convolutional capsules in Digit Caps consist of three layers: two of the 32 channels consist of 8D capsules, while one of the three channels consists of 16D capsules for categorizing the segmented mask. The number of neurons is indicated by the symbol “D”.

### U-Net architecture

Several popular segmentation networks include MTMC-AUR2CNet^46^, CMUNeXt^47^, DDA-SSNets^48^, SEA-NET^49^, and U-Net. MTMC-AUR2CNet’s design for multi-task learning and cross-consistency adds complexity, hindering practical deployment and rapid execution. CMUNeXt’s performance relies heavily on parameter configuration, requiring careful tuning of large kernels and skip fusion blocks for optimal results. DDA-SSNets, with their dual decoder attention mechanism, demand significant computational resources and fine-tuning, limiting their generalizability and usability. SEA-NET’s additional layers and attention modules increase model complexity and size, necessitating larger datasets to prevent overfitting and complicating interpretability. In contrast, U-Net’s streamlined encoder-decoder architecture offers ease of implementation, reproducibility, and efficient performance on standard hardware. U-Net’s relatively simple design allows it to be more generalizable and accessible compared to other models, which often require fine-tuning and specialized hardware for optimal performance. This makes the U-Net a more practical choice for broader applications, particularly in clinical settings where rapid deployment and reliable outcomes are critical. The U-Net model used in this study has been optimized to work with the Conv-Caps classifier. This tailored optimization enhances the overall model performance by ensuring compatibility and efficient data flow between the segmentation and classification stages. The following modifications have been made to the traditional U-Net architecture:

- The U-Net architecture features an encoder-decoder structure that captures multi-scale context information. The enhanced version integrates capsule networks within this framework to maintain spatial hierarchies more effectively.
- Skip connections are employed between the encoder and decoder paths. These connections ensure that high-resolution features from the encoder are directly accessible to the decoder, aiding in precise localization during segmentation.
- To improve segmentation accuracy, a hybrid loss function that combines dice coefficient loss and cross-entropy loss is used. This approach balances the need for pixel-wise accuracy and the overall segmentation quality.
- An attention mechanism is integrated into the U-Net to focus on the most relevant parts of the feature maps. This mechanism helps the model to better differentiate between the regions of interest and the background, leading to more accurate segmentation results.

### Severity detection

**Figure 4:**
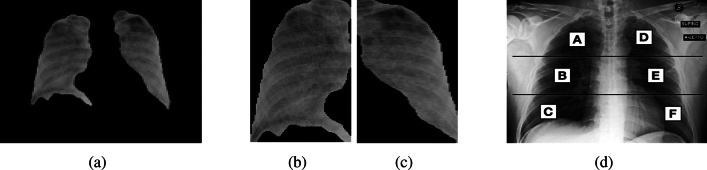
Different phases in lung Image Severity assessment.

The severity assessment of CXR scans is estimated by utilizing a bounding box derived from the segmented mask. The labels assigned to the scans encompass viral pneumonia, normal status, and COVID-19 infection. However, for individuals with COVID-19-affected CXR images, cases of normal and pneumonia states are excluded. The primary focus is on identifying severity within the segmented lung images. To achieve this, the segmentation mask is utilized to arrange contours based on their respective areas, Fig. 4a shows the image after applying the segmentation mask. The two largest area contours from the segmented lung mask are then cropped, representing the left and right lung sections in case of contamination as shown in Fig. 4b and c respectively. These lung regions are further divided into three segments (upper, middle, and lower) following the Brixia score system, each assigned a score ranging from 0 to 3 based on observed pulmonary infiltrate categories.

These segments are then split into vertically distinct sub-segments labeled as (A, B, C) and (D, E, F) for finer categorization as shown in Fig. 4d. The upper area (A and D) corresponds to the inferior wall of the aortic arch, the middle region (B and E) spans from the inferior wall of the aortic arch up to the inferior wall of the right inferior pulmonary vein, and the lower area (C and F) lies below the inferior wall of the right inferior pulmonary vein.

The severity scoring is defined as follows: 0 for no lung abnormalities, 1 for interstitial infiltrates, 2 for dominant interstitial infiltrates with some alveolar involvement, and 3 for dominant interstitial and alveolar infiltrates. Utilizing multiple DL models, the optimal severity score for CXR images is predicted. The loss function acts as a fundamental measure to quantify the disparities between the predicted severity scores and the actual labels obtained through various DL models, including ResNet-18, VGG-16, and DenseNet-201. By employing metrics such as mean absolute error (MAE) and mean squared error (MSE) for performance evaluation, a more comprehensive understanding of the model’s performance is provided. Such elaboration will not only elucidate its significance in optimizing severity predictions but also contribute to the overarching enhancement of the framework’s efficacy.

## Performance evaluating measures

The proposed framework is composed of different modules, each with its specific function. To gauge the efficacy of these modules, a diverse set of accuracy metrics has been employed for performance evaluation. The effectiveness of the U-Net segmentation model is assessed by employing dice loss (also called the Sørensen-dice index), Intersection over union (also known as Jaccard index), precision, and recall metrics. A frequent loss function in semantic segmentation problems is dice loss ($\mathscr{D}\mathscr{L}$). It is determined by computing the dice coefficient between the anticipated and ground truth segmentation masks for each pixel and then averaging these values across all pixels. The formulation of the $\mathscr{D}\mathscr{L}$ is as follows:

$$\begin{aligned} \mathscr{D}\mathscr{L} = 1 - 2\times \left( \frac{ \mathscr {G} \cap \mathscr {G}_{pred} + \xi }{\mathscr {G} + \mathscr {G}_{pred} + \xi }\right) \end{aligned}$$

where the intersection is the number of pixels accurately classified in both the anticipated and ground truth masks and the sum of the predicted $(\mathscr {G}_{pred})$ and ground truth $\mathscr {G}$ is the entire amount of pixels in each mask. $\mathscr{D}\mathscr{L}$ represents the dice loss of the models and varies from $\mathscr{D}\mathscr{L} \in [0, 1]$. In order to prevent division by zero, a minor constant ${\xi }=1$ is linked to the numerator and denominator. Furthermore, intersection over union (IoU) is employed to evaluate object detection effectiveness by contrasting the ground truth bounding box to the predicted bounding box. It can be determined as:

$$\begin{aligned} IoU= \frac{\mathscr {I}_{AOO}}{ \mathscr {I}_{AOU}} \end{aligned}$$

where the areas of overlapping and union are denoted by $\mathscr {I}_{AOO}$ and $\mathscr {I}_{AOU}$, respectively. The ranges of IoU exist between 0 to 1. This study gauges the classification model’s performance in terms of sensitivity (recall), specificity, accuracy, $\mathscr {F}_{1}{Score}$, and precision (also known as positive predictive value or PPV). The proportion of accurately detected positives and negatives is measured by sensitivity and specificity. Accuracy measures the percentage of both positive and negative instances that are accurately classified. The standard statistical metrics are also computed:

$$\begin{aligned}\mathscr {Y}_{\mathscr {R}}&= \frac{\mathscr {T}_p}{\mathscr {T}_{p} + \mathscr {F}_{n}}\\ \mathscr {Y}_{\mathscr {P}}&= \frac{\mathscr {T}_{p}}{\mathscr {T}_{p} + \mathscr {F}_{p}}\\ \mathscr {Y}_{\mathscr {S}}&=\frac{\mathscr {T}_{n}}{\mathscr {T}_{n}+\mathscr {F}_{p}}\\ \mathscr {Y}_{\mathscr {A}}&= \frac{\mathscr {T}_{n} + \mathscr {T}_{p}}{\mathscr {T}_{n} + \mathscr {T}_{p} + \mathscr {F}_{p} + \mathscr {F}_{n}} \end{aligned}$$

where $\mathscr {F}_{p}$ and $\mathscr {F}_{n}$ stand for false positives and false negatives, while $\mathscr {T}_{p}$ and $\mathscr {T}_{n}$ indicates the true positives and true negatives of the models, respectively.

Moreover, $\mathscr {F}_{1}{Score}$ is the harmonic mean of a model’s precision and recall values. It can be stated that way:

$$\begin{aligned} \mathscr {F}_{1}{Score} = \frac{2 \times (\mathscr {Y}_{\mathscr {P}} \times \mathscr {Y}_{\mathscr {R}}) }{\mathscr {Y}_{\mathscr {P}} + \mathscr {Y}_{\mathscr {R}}} \end{aligned}$$

where $\mathscr {Y}_{\mathscr {P}}, \mathscr {Y}_{\mathscr {R}}, \mathscr {Y}_{\mathscr {S}}$, and $\mathscr {Y}_{\mathscr {A}}$ stood for the model’s precision, recall (sensitivity), specificity, and accuracy. The range of $\mathscr {Y}_{\mathscr {P}}$ and $\mathscr {Y}_{\mathscr {R}}$ varies from 0 to 1.

The study assessed the efficacy of the severity detection methods by contrasting the MAE and MSE. A model assessment metric frequently used in relation to regression models is the MAE^50^. Each prediction error represents the difference between the model’s real and anticipated values. It can be formulated as follows:

$$\begin{aligned} \mathscr {Y}_{MAE} = \frac{1}{\Lambda } \sum _{j=1}^{\Lambda }\left( \mathscr {J}_{j} - \hat{\mathscr {J}_{j}}\right) \end{aligned}$$

MSE is a quadratic measure because it squares error, resulting in larger weights for significant errors than MAE. The explanation of MSE is as follows:

$$\begin{aligned} \mathscr {Y}_{MSE} = \frac{1}{\Lambda } \sum _{j=1}^{\Lambda }\left( \mathscr {J}_{j} - \hat{\mathscr {J}_{j}}\right) ^2 \end{aligned}$$

where $\mathscr {J}_{j}$ and $\hat{\mathscr {J}_{j}}$ indicate the real and anticipated values, respectively. The number of predictions is represented by $\Lambda$, which is finite.

Confidence interval is a statistical range, with a specified probability, that is likely to contain the true value of an unknown population parameter. It provides an estimated range of values which is likely to include the parameter, calculated from a given set of sample data. It can be formulated as follows:

$$\begin{aligned} CI = \bar{x} \pm z \left( \frac{s}{\sqrt{n}}\right) \end{aligned}$$

where $\bar{x}$ is the sample mean, *z* is the z-value from the standard normal distribution for a 95% confidence level (approximately 1.96), *s* is the sample standard deviation and *n* is the sample size.

### Ethical approval

The research work described in this manuscript does not require ethical approval, as it does not involve human subjects, animals, sensitive data, or any other aspect that falls under the purview of ethical or regulatory review.

## Experimental results and discussion

Extensive experiments were carried out using the CXR datasets as detailed in section “Dataset”. These experiments were conducted on the Google Colaboratory platform, utilizing Python 3.7 and a single GPU cluster equipped with an NVIDIA K80 GPU. This GPU configuration offers 12 GB of RAM and operates at a clock speed of 0.82 GHz. The CXR images were divided into three separate categories for training, validation, and testing, with proportions of 80%, 10%, and 10%, respectively. The complete objective has been accomplished through distinct modules. The initial step involves creating a U-Net model to generate detailed segmentation masks for lung regions. U-Net architecture takes advantage of modern GPU capabilities, allowing for efficient categorization of CXRs. The fundamental principle behind this implementation is to strategically employ progressively smaller layers paired with up-sampling operators to attain higher-resolution outcomes from the input CXR. The U-Net model’s computational complexity arises from its encoder-decoder architecture, involving several convolutional layers, pooling, and up-sampling operations. Despite this, the model was optimized for efficiency, completing the training process in 100 epochs with a learning rate of 0.001. Various learning rates were tested, and 0.001 offered the best balance between convergence speed and model performance. The Adam optimizer was used for its efficiency and ability to handle sparse gradients in noisy problems. Training for 100 epochs was sufficient, as indicated by the observed training and validation loss curves.

**Table 2:** Assessment of U-Net segmentation model performance.

Performance evaluation	Values
$\mathscr{D}\mathscr{L}$	0.0221
*IoU*	0.9790
$\mathscr {Y}_{\mathscr {R}}$	0.9903
$\mathscr {Y}_{\mathscr {P}}$	0.9924

The effectiveness of the U-Net segmentation model is highlighted in Table 2 through various metrics. The model is designed to generate segmentation masks that closely resemble the actual objects present in the lung image. $\mathscr{D}\mathscr{L}$ is particularly advantageous for datasets with imbalanced class distributions, where certain classes might be underrepresented. It prioritizes the training of these less common classes, ensuring that they are not neglected during the optimization process. The data in Table 2 demonstrates a relatively low dissimilarity (as indicated by $\mathscr{D}\mathscr{L}$) between the predicted and ground truth segmentations. This suggests that the U-Net segmentation model effectively classifies pixels with accuracy. The IoU score indicates that the predicted segmentation closely resembles the ground truth segmentation, signifying high precision. Furthermore, the recall ($\mathscr {Y}_{\mathscr {R}}$) metric measures the model’s ability to distinguish true positive instances (pixels belonging to the target class) from all other positive instances present in the ground truth. The U-Net model shows strong recall, indicating that it detected a substantial number of positive instances. Precision, on the other hand, measures the model’s capacity to accurately identify true positive cases among all instances predicted as positive. A precision value of 0.9924 signifies outstanding model performance in predicting positive instances while minimizing false positives. These evaluation metrics thoroughly demonstrate the U-Net model’s exceptional performance in accurately segmenting the target class. The model achieves a high level of similarity, accuracy, and captures a significant portion of positive instances.

In the second module, the Conv-Caps is utilized to differentiate between three categories: normal, pneumonia, and COVID-19 instances, based on bounded box data. The dataset is partitioned into 70% for training, 15% for validation, and 15% for testing purposes. A learning rate of 0.001 is chosen after testing several values, ensuring stable convergence and effective training. The Conv-Caps network, despite its computational complexity due to the dynamic routing algorithm, showed significant improvement in classification accuracy. The model was trained for 85 epochs with a batch size of 10, with these values fine-tuned using a grid search to systematically evaluate performance across a range of values. The utilization of heatmaps provides a visual representation of data using distinct colors to indicate varying values or intensities.

**Figure 5:**
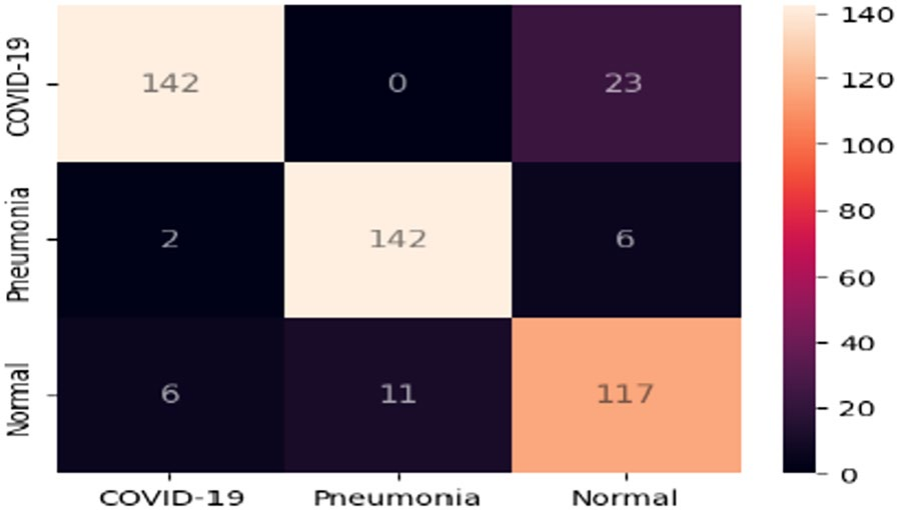
Conv-Caps confusion matrix for classifying Normal, Pneumonia or COVID-19.

*Detection rate analysis:* A significant detection rate is essential for COVID-19 screening. The proposed Conv-Caps model demonstrated effective performance based on the provided confusion matrix in Fig. 5. The model achieved a high true positive rate of 86% for COVID-19 cases and 14% false negative rate. For Pneumonia, the model showed a true positive rate of 93% and a false negative rate of 7%. For Normal cases, the model achieved a true positive rate of 85% and a false positive rate of about 15%. These results highlight the model’s capability to significantly reduce false positives and false negatives, ensuring high precision (ranging from 93-98%) and reliable screening. This reduction in misclassification is crucial for alleviating the burden on healthcare systems by minimizing the number of healthy individuals or non-COVID-19 patients falsely diagnosed with COVID-19.

**Table 3:** Performance comparison of the proposed model and other classifiers on the test set.

Models	$\mathscr {Y}_{\mathscr {A}}$	$\mathscr {Y}_{\mathscr {S}}$	$\mathscr {Y}_{\mathscr {P}}$	$\mathscr {F}_{1}{Score}$
Covid-Net	93.30	93.33	93.56	93.48
VGG-19	83.00	82.33	85.50	83.74
Resnet-50	90.60	90.66	91.20	90.94
Conv-Caps	93.98	93.99	93.97	93.98

The model’s performance metrics, including precision, recall, and the $\mathscr {F}_{1}{Score}$, are presented in Table 3, alongside a comparison with other baseline classifiers. The Conv-Caps model demonstrated impressive accuracy across various sets: 97.48% on the training set, 94% on the validation set, and 93.98% on the testing set. In the COVID-19 category (column), precision (positive predictive value) stands at 93.97%, indicating the proportion of accurate COVID-19 predictions out of all predicted cases. Recall signifies the percentage of actual cases correctly identified by the model, with a recall of 93.99%. The $\mathscr {F}_{1}{Score}$, at 93.98%, demonstrates a balanced trade-off between precision and recall, showcasing the model’s robustness.

**Figure 6:**
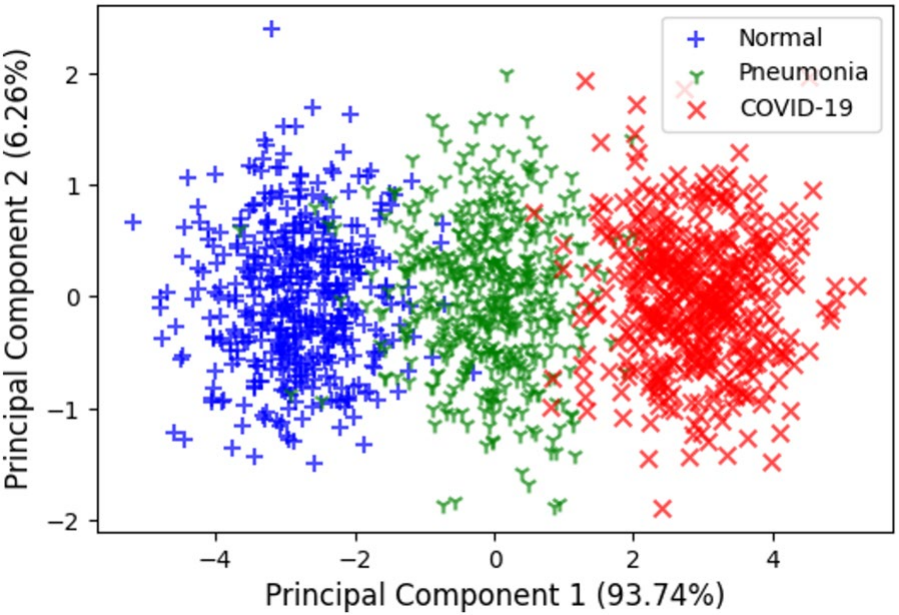
Feature visualization for the Conv-Caps on the test set.

*Feature space-based analysis:*An analysis of the feature space learned by the Conv-Caps model is carried out to gain a deeper understanding of decision-making behavior. The good discrimination ability of a classifier is generally associated with the characteristics of the feature space. Class-distinguishable features improve learning and reduce the model’s variance on diverse inputs. Visualization of the feature space is performed by plotting the principal components of the data. Figure 6 shows the 2-D plots of principal component 1 and principal component 2, along with their percentage variance, for the proposed Conv-Caps models for the test set. Data plotting reveals that the Conv-Caps model effectively separates the classes of Normal, Pneumonia, and COVID-19 cases. While some overlap exists between the Normal and Pneumonia cases, the majority of data points for each class are well-clustered, indicating distinct feature spaces for each class. This clear separation suggests that the Conv-Caps model has a strong capability for feature extraction and class discrimination, contributing to its robust classification performance. The principal component analysis (PCA) demonstrates that the feature space learned by the Conv-Caps model is diverse and well-structured, which enhances its ability to distinguish between different classes.

*Confidence interval analysis:* To evaluate the measurement uncertainty of our models, we calculated the 95% confidence intervals for the key performance metrics of the proposed Conv-Caps model. This analysis was performed using a sample size of 491, with a standard deviation of 1.5. The 95% confidence intervals for the performance metrics of the Conv-Caps model are as follows: accuracy [93.848, 94.112], sensitivity [93.858, 94.122], precision [93.838, 94.102], and $\mathscr {F}_{1}{Score}$ [93.848, 94.112]. These intervals indicate that we can be 95% confident that the true values for all the performance measures lie within the specified ranges, demonstrating the reliability and robustness of the model’s performance.

The next module explores the severity prediction with a particular emphasis on CXR images of COVID-19-detected patients. This endeavor seeks to utilize the potential of advanced ML techniques to identify the severity of the infection, offering an approach to help in well-informed medical decision-making. In the context of severity prediction derived from segmented masks, a crucial step involves the extraction of a bounding box. The CXRs consist of areas beyond the lungs, which are not relevant to the focus of our study. This could potentially lead to the model learning features that are not pertinent to the study’s objectives. To overcome this, an algorithm based on anatomical atlases^51^ is employed to automatically identify the region of interest (ROI), which is the lung area in this case. To achieve this, a reference set of CXRs from patients, with lung masks expertly delineated, is utilized as the models^52^. These models are then aligned with the target CXRs being analyzed. The alignment process involves establishing correspondence between the features of the target CXR and the model CXRs using the SIFT-flow algorithm^53^. This correspondence helps transform the model lung masks to closely match the lung structure in the target CXR, essentially creating an approximate lung model. The boundaries of these aligned lung models are then cropped to form a bounding box that encapsulates all the lung pixels relevant to the current task, ensuring a focused region of interest for analysis.

**Table 4:** Models assessment based on MAE.

Models	$\mathscr {Y}_{MAE}$					
	A	B	C	D	E	F
ResNet50	0.850	0.851	0.795	0.843	0.791	0.690
VGG-16	0.925	0.948	0.932	0.970	0.894	0.883
DenseNet 201	0.465	0.756	0.736	0.870	0.498	0.680

In the subsequent severity prediction, a comprehensive strategy is adopted where all six distinct parts of the CXR images are effectively employed. The dataset is divided into a training set comprising 80% of the data and a testing set containing the remaining 20% for comprehensive evaluation. This partitioning ensures both robust performance assessment and a balanced representation of data in training and testing. For the severity prediction models (ResNet50, VGG-16, DenseNet201), a fixed learning rate of 0.001 was selected based on preliminary experiments that indicated stable learning. Each model was trained for 100 epochs with a batch size of five to manage memory constraints and provide sufficient updates per epoch. An early stopping mechanism and learning rate reduction upon a plateau in validation loss for five epochs were employed to prevent overfitting and allow finer adjustments in later training stages. While DenseNet201, being more complex due to its densely connected layers, provided superior performance with lower MAE and MSE values, all models demonstrated effective training as evidenced by the validation loss.

**Figure 7:**
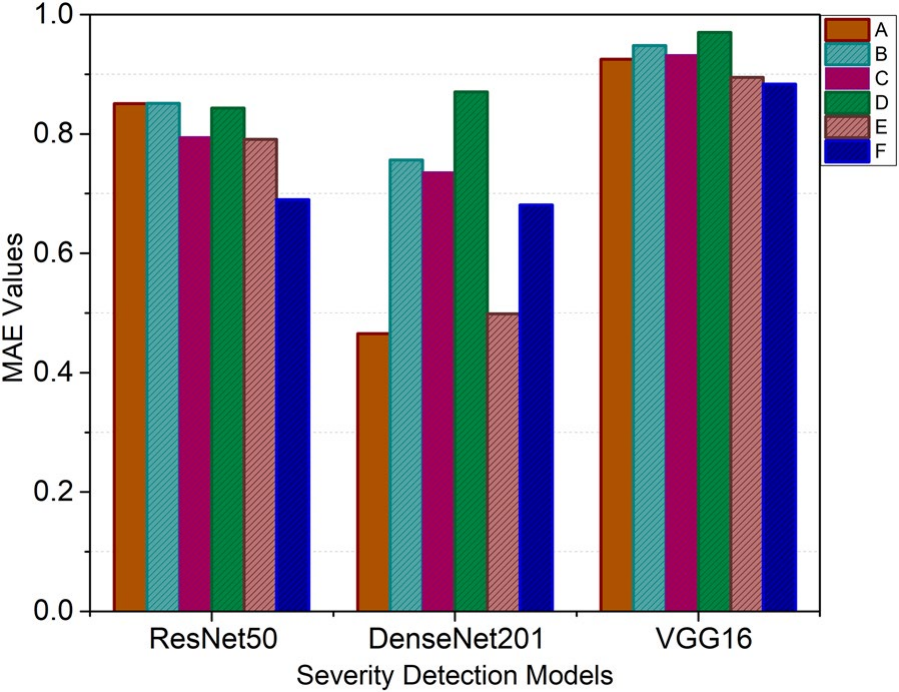
MAE values for severity detection.

*Performance analysis:* The DenseNet201 model outperformed the other models in predicting the severity of lung infection across all segmented regions (A, B, C, D, E, F). The MAE values for each model are presented in Table 4, while Fig. 7 displays the models’ performance graphically. DenseNet201 consistently shows lower MAE values compared to ResNet50 and VGG-16. This indicates that DenseNet201 provides more accurate predictions, capturing the intricate features of the segmented lung regions more effectively. While ResNet50 demonstrated respectable performance, its MAE values were higher than those of DenseNet201, indicating less precise predictions. VGG-16 showed the highest MAE values among the three models, suggesting it was the least accurate at predicting severity. DenseNet201 achieved the lowest MAE values across all regions, highlighting its superior ability to predict severity accurately. Table 4 presents the recorded MAE values for each segmented mask region (A, B, C, D, E, F) for the studied models.

**Figure 8:**
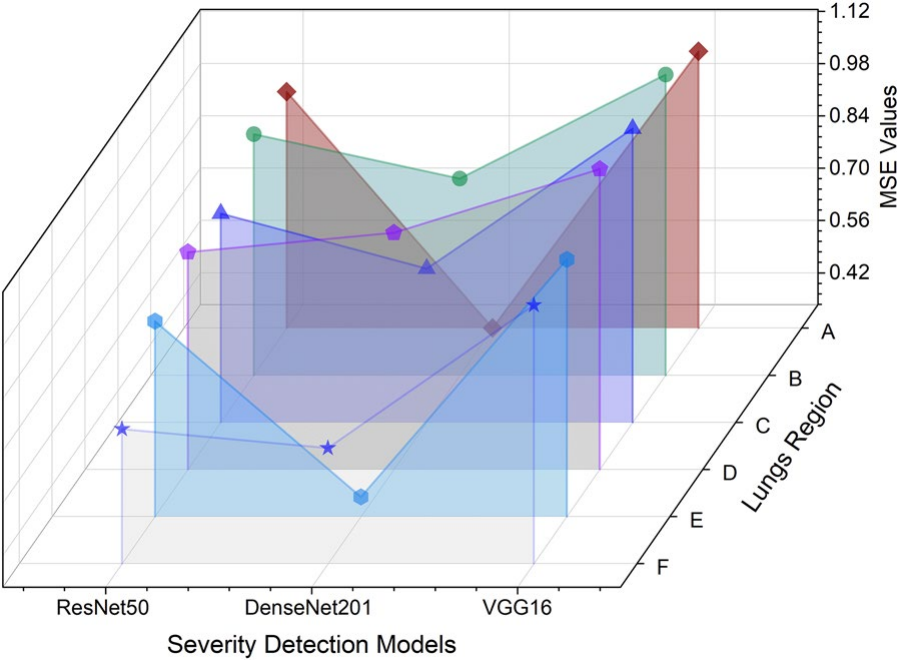
Model’s performance evaluation in severity prediction across different lung regions.

The comparative evaluation of three alternative ML models across various categories is facilitated by Table 5, where their respective MSE values are utilized. Notably, the MSE scores associated with the DenseNet201 model reflect enhanced efficacy in assessing the severity of lung region detection within CXR images. In the context of this assessment, lower MSE scores imply a higher level of performance. Figure 8 visually presents the comparison of the model’s performance in severity detection for distinct lung regions.

**Table 5:** Analysis of the severity detection based on MSE values.

Models	$\mathscr {Y}_{MSE}$					
	A	B	C	D	E	F
ResNet50	0.9678	0.9804	0.8938	0.9164	0.8750	0.6950
VGG-16	1.0761	1.1396	1.1211	1.1391	1.0231	1.0268
DenseNet 201	0.3351	0.8612	0.7463	0.9684	0.3874	0.6442

*Detailed observations:* The infection levels in lung regions E and F were relatively lower, as indicated by the lower severity scores. This trend was consistently captured by DenseNet201, showcasing its precision. Lung regions D and B exhibited more pronounced infection levels, with higher severity scores. DenseNet201 accurately identified these areas of severe infection, which is critical for clinical decision-making.

*Practical implications:* The ability of DenseNet201 to consistently provide accurate severity predictions has significant practical implications. Accurate severity assessment can guide clinicians in making informed decisions regarding patient management, treatment planning, and resource allocation. The comprehensive analysis highlights DenseNet201’s superior capability in predicting the severity of COVID-19 infection from CXR images. By consistently achieving lower MAE and MSE values, DenseNet201 proves to be a reliable tool for severity assessment, which is crucial for effective patient care and management in clinical settings.

The proposed approach has some limitations. Firstly, the segmentation accuracy, while generally high, can still suffer from inaccuracies that adversely impact the subsequent classification and severity prediction stages. Any errors in lung region segmentation could lead to less reliable results. Secondly, the model’s ability to generalize to other respiratory conditions is limited. Designed specifically for COVID-19 severity detection, it may not perform as well for other diseases without further training and adaptation. Additionally, interpretability remains a significant challenge. DL models often function as “black boxes,” making it difficult to understand the decision-making process, which is crucial in medical applications. Finally, the approach’s computational demands and complexity may hinder its application in real-time or resource-constrained environments, limiting its practicality for rapid diagnosis. These limitations suggest areas for future improvement to enhance the robustness and applicability of the proposed approach.

## Conclusion and future work

The presented framework combines ML and DL techniques to successfully identify COVID-19 infections and categorize their severity levels based on CXR images, contributing to early detection and informed medical decision-making. By leveraging the Brixia score assessment, the severity categorization gains clinical significance and aids in patient care. The research encompasses three modules, each customized for a specific task. The U-Net model showcases exceptional performance in segmenting lung regions within CXR images, effectively generating precise segmentation masks. The subsequent module employs the Conv-Caps model for classification tasks, distinguishing between normal, pneumonia, and COVID-19 cases based on bounding box data. The Conv-Caps model exhibits commendable accuracy across training, validation, and testing sets, ensuring robust classification. In the third module, meticulous segmentation and bounding box extraction are used to predict the severity of lung infections in COVID-19 patients. DenseNet201 emerges as a standout performer, delivering enhanced efficacy in detecting severity levels. This is evident through the lower MAE and MSE scores associated with the DenseNet201 model compared to other studied models. The graphical representation of these scores highlights the model’s superiority in severity detection across different lung regions. It is evident from this research that ML and DL technologies can significantly enhance our abilities to diagnose, classify, and predict infection severity. The proposed framework presents a valuable tool in combating the pandemic, aiding in efficient testing, accurate diagnosis, and informed patient care. Furthermore, the comparative assessment of different models offers insights into their performance, guiding future advancements in the field of COVID-19 detection and severity assessment using radiological imaging.

Future work can be focused on optimizing the framework for real-time processing, considering factors like computational efficiency and minimal latency, to enable timely and accurate decision-making. Deploying the developed model in real-time clinical settings might be a crucial step toward its practical applicability. Furthermore, expanding the dataset used for training and validation to include a more diverse range of patients, demographics, and disease manifestations can enhance the model’s generalization capability.

## Data Availability

The datasets analyzed during the current study are available at^54–57^.
